# Multiscale Investigation of the Factors Governing Ice–Asphalt Interfacial Adhesion Strength: Insights from Pull-Off Tests and Molecular Simulations

**DOI:** 10.3390/ma19132929

**Published:** 2026-07-07

**Authors:** Teng Yuan, Yunhao Jiao, Qian Su, Yujin Yao, Huaxin Chen, Yongchang Wu

**Affiliations:** 1 School of Materials Science and Engineering, Chang’an University, Xi’an 710064, China; tyuan@chd.edu.cn (T.Y.);; 2 Xinjiang Transport Planning Survey and Design Institute Co., Ltd., Urumqi 830006, China; 3 Xinjiang Key Laboratory for Safety and Health of Transportation Infrastructure in Alpine and High-Altitude Mountainous Areas, Urumqi 830006, China; 4 The Engineering Design Academy of Chang’an University Co., Ltd., Xi’an 710064, China; solusek201@outlook.com

**Keywords:** asphalt, ice adhesion, molecular dynamics simulation, interfacial interaction

## Abstract

Under low-temperature and high-humidity conditions, stable ice layers readily form on asphalt pavements in cold regions, and the enhanced ice–asphalt interfacial adhesion significantly increases deicing difficulty and traffic safety risks. To clarify the factors governing ice–asphalt interfacial adhesion strength, this study combines macroscopic pull-off tests and molecular dynamics simulations to systematically investigate the effects of interfacial contact area, temperature, pull-off rate, and molecular characteristics of representative asphalt components. The pull-off results show that adhesion strength increases markedly with decreasing temperature, rising from approximately 163 kPa at −2 °C to 242 kPa at −10 °C. In contrast, the nominal adhesion strength decreases with increasing ice specimen size, suggesting that size-related interfacial heterogeneity and nonuniform stress transfer may contribute to the pull-off response. The adhesion strength also generally decreases as the pull-off rate increases. Molecular dynamics simulations show that smaller asphalt–ice interfacial models exhibit higher molecular-scale nominal adhesion responses, while temperature-dependent simulations provide short-range asphalt–ice interaction descriptors for interpreting the experimental temperature trend. The calculated short-range asphalt–ice interaction energy becomes less negative from −531.4 to −352.5 kJ mol^−1^ with increasing temperature, supporting the experimentally observed strengthening of adhesion at lower temperatures. Single-molecule pull-off simulations of 12 representative asphalt molecules reveal pronounced molecular differences, with molecular-scale nominal adhesion strengths ranging from 303.7 to 734.6 MPa. Asphaltene and polar aromatic molecules generally show stronger adhesion, which is associated with larger projected contact area, flatter molecular configurations, and heteroatom-induced polar sites. The molecular polarity index shows a moderate positive association with molecular-scale nominal adhesion strength. These results establish a scale-aware mechanistic correspondence between macroscopic pull-off behavior and molecular interaction descriptors at the ice–asphalt interface, providing insights for interfacial adhesion regulation and anti-icing design of asphalt pavement materials in cold regions.

## 1. Introduction

In cold regions, transportation infrastructures such as roads, bridge decks, and airport pavements are prone to icing under low-temperature and high-humidity conditions [1,2,3,4]. Ice accretion markedly reduces surface skid resistance, increases braking distance and operational risk, and ultimately undermines winter traffic safety and maintenance efficiency. For asphalt pavements, however, the risk associated with icing is governed not only by ice formation itself, but also by the interfacial adhesion between ice and the asphalt substrate. As the ice–asphalt interfacial adhesion strength increases, the ice layer becomes more difficult to remove from the pavement surface, and the energy demand for deicing rises accordingly. Therefore, ice–asphalt interfacial adhesion strength has been widely regarded as a key indicator for evaluating pavement icing behavior, deicing difficulty, and interfacial anti-icing performance [5,6,7].

Current engineering strategies for mitigating pavement icing mainly include mechanical deicing, deicing salt application, and the development of anti-icing surfaces. Although salt-based deicing is widely practiced, its long-term use may cause corrosion of roads and bridges and raise environmental concerns [8,9]. By contrast, reducing ice–pavement interfacial adhesion to facilitate ice removal has attracted increasing attention as a material-level anti-icing strategy [10,11,12,13,14,15]. Previous studies have demonstrated that the measured ice–pavement adhesion strength is strongly affected by freezing temperature, surface texture, and specimen geometry. Nevertheless, variations in test setups and geometric configurations often result in substantial discrepancies among reported results [1,16,17], indicating that macroscopic test data alone are still insufficient to distinguish how contact size, temperature, pull-off rate, and molecular-level interfacial interactions jointly affect ice–asphalt adhesion.

In recent years, molecular dynamics (MD) simulation has become an important molecular-scale approach for investigating the interfacial behavior of asphalt materials [18,19,20,21,22,23,24]. It has been extensively employed to investigate asphalt composition, aging, adhesion, and diffusion, offering molecular-level insights into the evolution of macroscopic performance [21,22,23,24,25,26,27,28,29]. For asphalt–ice interfaces, recent studies have begun to examine the quasi-liquid layer (QLL), interfacial hydrogen bonding, and molecular polarity as molecular descriptors related to freeze-adhesion behavior [30,31,32,33,34,35]. These studies indicate that temperature-dependent ice-surface structure and interfacial molecular polarity can influence asphalt–ice adhesion, but the relative contributions of contact geometry, loading condition, and asphalt molecular composition remain insufficiently resolved [30,31,32,33,34,35]. Despite these advances, MD studies that directly address pull-off failure at the asphalt–ice interface and connect molecular descriptors with macroscopic adhesion trends remain limited.

Moreover, asphalt is a complex multicomponent organic binder composed of molecular fractions that differ in size, configuration, aromaticity, planarity, polarity, and heteroatom-containing functionalities [18,24,25]. Their contributions to adhesion at an ice surface are therefore unlikely to be identical. Previous molecular-scale studies on asphalt-related interfaces have suggested that molecular geometry, aromatic framework, atomic density, and heteroatom distribution can influence interfacial adhesion. This insight suggests that freeze-adhesion behavior at the ice–asphalt interface may likewise be closely associated with the geometric configuration and polarity distribution of representative asphalt molecules. However, for asphalt–ice interfaces, the single-molecule pull-off behavior of representative asphalt molecules, their projected contact areas, surface electrostatic-potential distributions, and quantitative polarity–adhesion relationships remain insufficiently established. This limitation hinders a deeper understanding and regulation of ice–asphalt freeze adhesion from the perspective of material composition.

Based on the above considerations and building on our previous work [35,36], this study combines macroscopic pull-off tests with molecular dynamics simulations to systematically investigate the multiscale mechanisms governing ice–asphalt interfacial adhesion strength. First, macroscopic pull-off tests were performed to examine the effects of temperature, ice specimen size, and pull-off rate on interfacial adhesion strength. Second, representative asphalt–ice interfacial simulations were constructed using the literature-established AAA-1 12-component asphalt model based on the Li and Greenfield framework [19,20]. This model was used as a representative multicomponent asphalt model for mechanistic comparison, not as a molecule-by-molecule reconstruction of the experimental binder. These simulations were used to evaluate how interfacial size and temperature affect nominal adhesion response and asphalt–ice interaction descriptors. Third, single-molecule pull-off simulations of 12 representative asphalt molecules on ice were combined with projected-area and molecular surface electrostatic-potential analyses to quantify molecular-level adhesion descriptors and evaluate the association between molecular polarity and molecular-scale nominal adhesion strength. Through this framework, the study distinguishes directly measured macroscopic pull-off trends, computed asphalt–ice interfacial descriptors, and molecular polarity/contact-area associations. The results are intended to provide mechanistic insight into ice–asphalt interfacial adhesion and to support future interfacial regulation strategies for asphalt pavement materials while recognizing that direct pavement-scale anti-icing performance requires further validation.

## 2. Molecular Dynamics Simulation and Experimental Section

### 2.1. Molecular Dynamics Simulation

#### 2.1.1. MD Simulation Procedure

**Figure 1:**
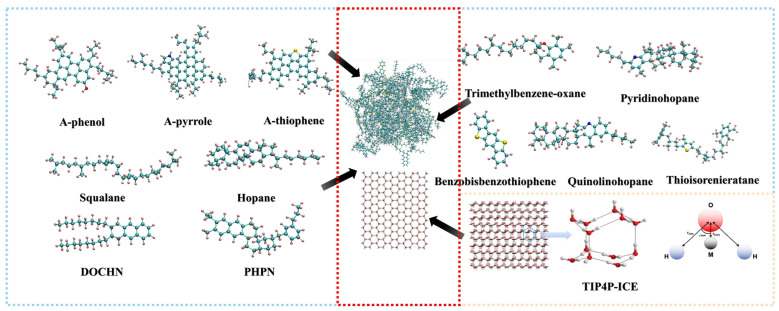
Schematic representation of the 12-component asphalt molecular model, the TIP4P-ICE water model, and the asphalt–ice interfacial model.

The asphalt phase was represented by the literature-established AAA-1 12-component asphalt model developed within the Li and Greenfield framework [19,20]. The model contains 12 molecular species representing the main asphalt fractions, including three asphaltene species, two naphthene aromatic species, five polar aromatic species, and two saturate species. This multicomponent composition provides a practical representation of asphalt chemical diversity while remaining computationally tractable for asphalt–ice interfacial simulations. Previous studies have reported that the simulated density and thermal expansion coefficient of this model are in reasonable agreement with experimental values, supporting its use as a representative asphalt model for molecular simulations. The molecular composition and selected structural descriptors of the representative AAA-1 model are summarized in Table 1. In addition, a hexagonal ice crystal (Ih) constructed using the TIP4P-ICE model [37] was selected as the ice substrate to establish the simplified single-molecule/ice-crystal and asphalt/ice-crystal models, as illustrated in Figure 1.

Water molecules were represented using the TIP4P-ICE water model [37]. This model, part of the TIP4P series, is renowned for its extensive use in MD simulations due to its ability to accurately mimic water’s phase diagram and efficiency in large-scale simulations. It is particularly favored for simulating the water–ice interface, given its precise rendering of ice lattice structures and melting temperatures [38]. The basic parameters of the TIP4P water model are listed in Table 2.

#### 2.1.2. MD Simulation Details

Molecular dynamics (MD) simulations were performed using GROMACS 2020.3 [39]. The OPLS-AA (Optimized Potentials for Liquid Simulations-All Atom) force field was employed throughout the simulations to define the potential energy functions, because it is highly compatible with the TIP4P water model and is suitable for simulations of organic and polymeric molecular systems [40]. Bond stretching and angle bending were described using harmonic potentials. Long-range electrostatic interactions were calculated using the particle mesh Ewald (PME) method [41,42], while short-range interactions were evaluated using the cutoff scheme defined in the simulation parameter file. Detailed nonbonded interaction settings are provided in the Supplementary Information. The LINCS algorithm [43] was applied to constrain bond lengths and maintain the bonded geometry during simulation. Temperature coupling was performed using the Nosé–Hoover thermostat with a time constant of 0.5 ps [44,45]. When pressure coupling was required, the Parrinello–Rahman barostat was adopted under the constant number of particles, pressure, and temperature (NPT) ensemble [46]. Atomic trajectories were integrated using the Verlet algorithm [47]. Periodic boundary conditions (PBCs) were applied according to the simulation setup, and for interfacial pull-off systems, a vacuum region was retained along the surface-normal z direction to reduce artificial interactions between periodic images during separation. To support model-stability assessment, additional 5 ns validation trajectories were analyzed for representative asphalt and asphalt–ice interface systems. The 0–1 ns segment was treated as the initial segment and the 1–5 ns window was used for quantitative summaries, as shown in Supplementary Figures S1a–S1c.

**Figure 2:**
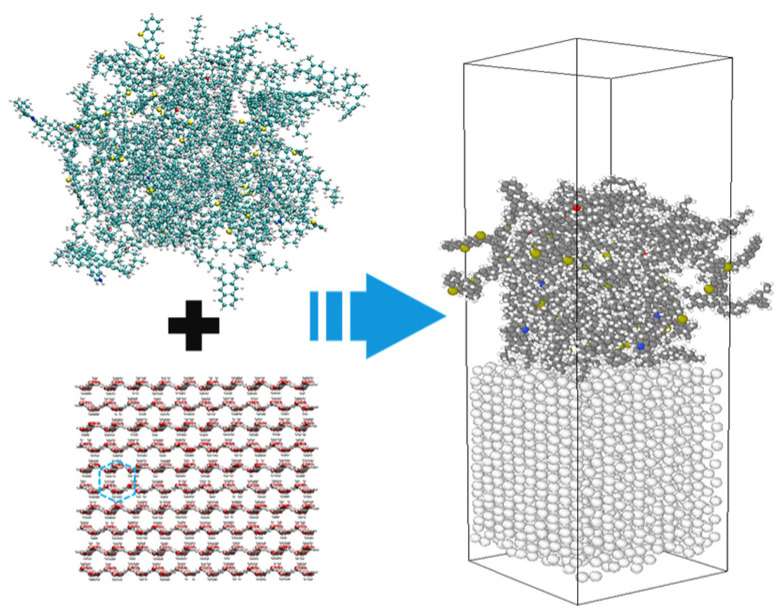
Schematic illustration of the construction of the asphalt–ice interfacial model from the asphalt phase and the Ih ice substrate.

A hexagonal ice crystal (Ih) with box dimensions of 50 Å × 40 Å × 55 Å was constructed from 2000 TIP4P-ICE water molecules. The asphalt phase was subsequently positioned above the ice surface to establish the asphalt–ice interfacial model, as illustrated in Figure 2. In addition, the bottom layer of the ice slab was fixed during pulling to prevent overall displacement of the substrate. Before pull-off, the model was equilibrated using the simulation settings described above, and additional 5 ns validation trajectories were used to assess model stability and interface-geometry maintenance, as shown in Supplementary Figures S1a–S1c. The adhesion characteristics of asphalt on the ice surface were analyzed through pull-off simulation.

**Figure 3:**
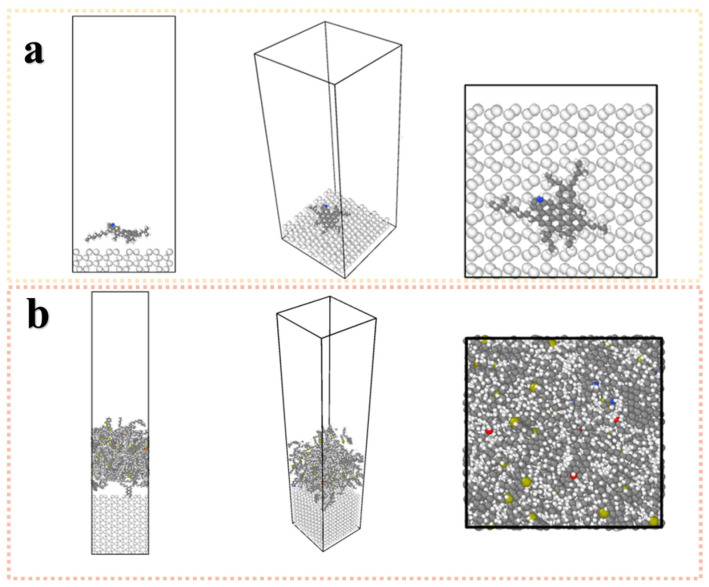
Initial configurations of the molecular models used in the pull-off simulations: (**a**) single-molecule/ice-crystal model and (**b**) asphalt–ice interfacial model. Side-view, perspective-view, and top-view snapshots are presented for each model.

Since the 12 representative asphalt molecules differ considerably in molecular structure and physicochemical characteristics, single-molecule pull-off simulations were conducted to clarify the differences in interfacial adhesion behavior among these characteristic molecules and to identify the corresponding controlling factors. The initial configurations of the models used in the pull-off simulations are shown in Figure 3, including the single-molecule/ice-crystal model (Figure 3a) and the asphalt–ice interfacial model (Figure 3b).

Following model construction, each representative asphalt molecule was first translated toward the ice surface at 0.001 nm/ps. When the closest intermolecular separation reached approximately 0.2–0.3 nm, corresponding to 2–3 Å, the approach step was stopped to generate a contacted initial configuration while avoiding obvious atomic overlap. The molecule was then pulled away from the ice surface at 0.001 nm/ps. The temperature was maintained at 260 K throughout the simulation. During the pull-off process, the interaction force between the asphalt molecule and the ice substrate and the molecular displacement were recorded to obtain the force–displacement response and maximum pull-off force. This pulling rate was used as a computationally tractable MD rate for relative single-molecule comparison and was not intended to match the laboratory pull-off rate.

### 2.2. Experimental Pull-Off Test of Ice–Asphalt Adhesion

**Figure 4:**
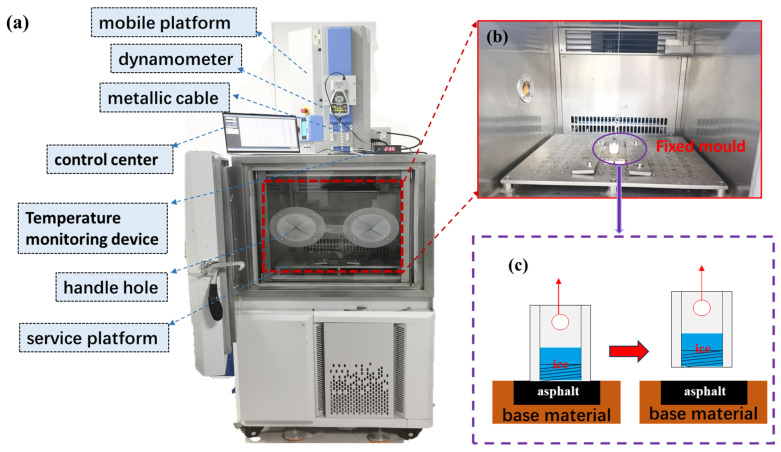
Experimental setup for the ice–asphalt interfacial pull-off test: (**a**) overall view of the integrated testing system, (**b**) fixed mould arrangement inside the environmental chamber, and (**c**) schematic illustration of the pull-off configuration.

Ice–asphalt interfacial adhesion strength was measured using a self-developed integrated low-temperature pull-off testing system, consisting mainly of environmental control, force measurement, software control, and test platform modules. The reliability of the apparatus was supported by previous PDMS-based calibration, parameter-sensitivity analysis, and comparison with literature data [36]. In that validation, the average ice–PDMS adhesion strength measured at −10 °C was 84 kPa, within the literature-reported range of 70–100 kPa. The apparatus configuration is shown in Figure 4, and the details of the validation procedure are provided in the previous experimental method study [36]. During testing, the maximum pull-off force required to detach ice from the asphalt surface was recorded, and ice–asphalt adhesion strength (IAS) was obtained by normalizing this peak force by the nominal ice–asphalt contact area; the calculation details are provided in the Supplementary Information. The standard test condition used a 20 mm ice diameter, corresponding to a nominal contact area of 3.14 cm^2^ and a pull-off rate of 40 mm/min. Each reported IAS value was averaged from at least 10 pull-off measurements. For the size-dependent tests, ice specimens with different diameters were prepared. For the rate-effect tests, additional pull-off rates within 10–50 mm/min were examined together with the standard 40 mm/min condition.

**Figure 5:**
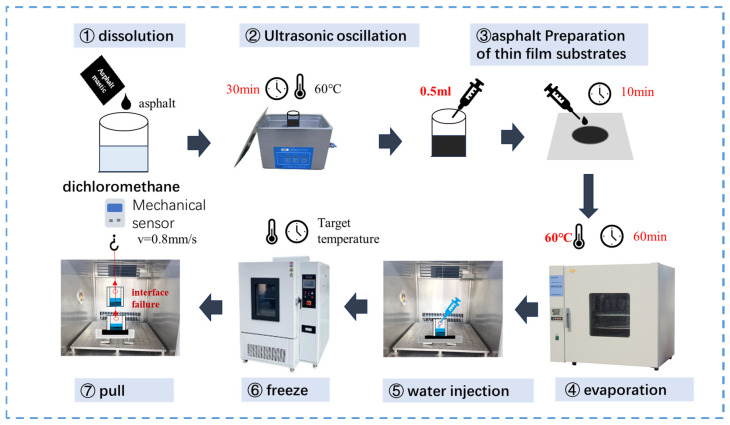
Schematic illustration of the experimental procedure, including asphalt dissolution, ultrasonic treatment, thin-film substrate preparation, solvent evaporation, water injection, freezing, and pull-off testing.

The preparation of the asphalt substrates and the detailed testing procedure for ice–asphalt adhesion are illustrated in Figure 5. Before testing, both the substrate and the ice mould were thoroughly cleaned using acetone (Aladdin Biochemical Technology Co., Ltd., Shanghai, China), ethanol (Aladdin Biochemical Technology Co., Ltd., Shanghai, China), and deionized water. The asphalt coating was prepared from HY90# asphalt (Shandong Jingbo Co., Binzhou, China) using the same procedure for all specimens to reduce variability associated with substrate preparation. After the target temperature was reached, the asphalt-coated substrate and the ice mould were placed in the environmental chamber and pre-cooled for approximately 30 min. Distilled water was then introduced into the mould positioned on the pre-cooled asphalt surface, followed by freezing at the target temperature for 60 min to form the ice specimen. During pull-off testing, the mould was connected to the dynamometer through a hook and metallic cable, and tensile force was applied vertically until detachment occurred. The force–displacement response was continuously recorded during loading. Before the formal pull-off test, the asphalt substrate, ice specimen, and loading fixture were kept at the target temperature for 1 h to promote thermal equilibration.

## 3. Results and Discussion

### 3.1. Effect of Interfacial Contact Area on Ice–Asphalt Adhesion Behavior

**Figure 6:**
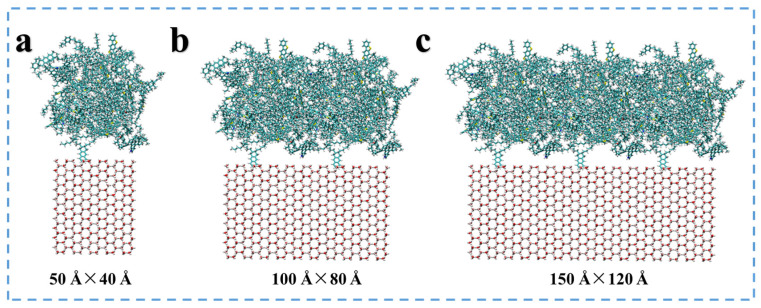
Snapshots of asphalt–ice interfacial molecular systems with three different interfacial sizes: (**a**) 50 Å × 40 Å, (**b**) 100 Å × 80 Å, and (**c**) 150 Å × 120 Å.

Molecular dynamics simulations indicate that the interfacial contact area and the number of interacting atoms are important factors governing the adhesion strength during pull-off at the ice–asphalt interface. To evaluate the size effect, three asphalt–ice interfacial models with different dimensions were constructed by varying the number of atoms in the interfacial system and the corresponding contact area, as shown in Figure 6.

**Figure 7:**
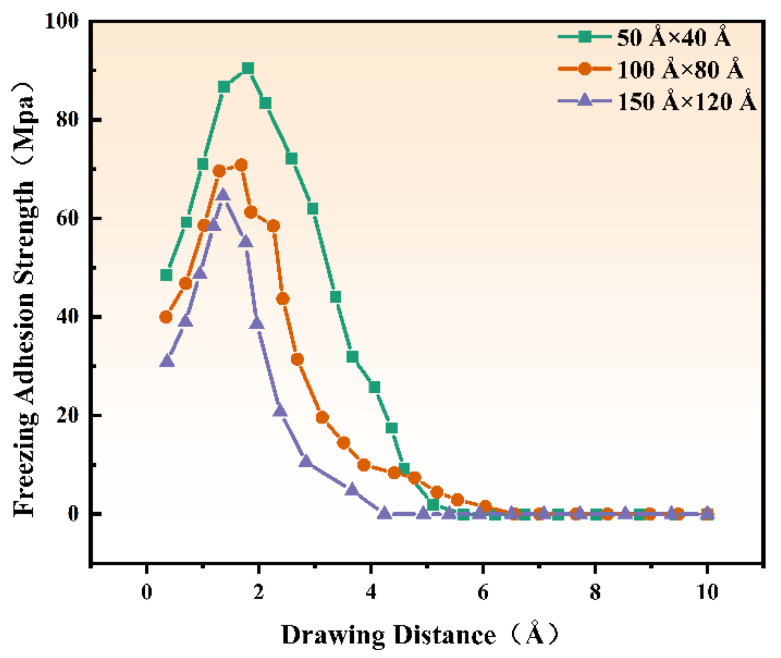
Adhesion strength as a function of pull-off distance for asphalt–ice interfacial models with different interfacial sizes.

As shown in Figure 7, the adhesion strength–pull-off distance curves of the asphalt–ice interfacial models with different interfacial sizes exhibit similar trends, characterized by a rapid increase at the initial stage, followed by a sharp decay after reaching the peak, and finally approaching zero. This indicates that the interface undergoes a process from initial elastic response and strong intermolecular interaction to gradual detachment and final separation. However, the peak adhesion strength is strongly dependent on interfacial size. As the interfacial size increases from 50 Å × 40 Å to 100 Å × 80 Å and 150 Å × 120 Å, the peak adhesion strength decreases from about 89 MPa to 70 MPa and 65 MPa, respectively, indicating that smaller interfacial models exhibit higher nominal adhesion strength in molecular dynamics simulations.

This behavior may be associated with finite-size effects and differences in interfacial stress transfer, although local stress fields and defect populations were not directly quantified in this work. In smaller interfacial models, the effective contact region is more spatially confined, and the pull-off load may be transferred through a relatively more coherent interfacial contact region, which is consistent with the higher peak nominal adhesion strength. By contrast, larger models may allow a greater likelihood of local structural heterogeneity and asynchronous detachment during pull-off, thereby reducing the area-normalized peak response. This interpretation is consistent with previous discussions of finite-size and heterogeneous interfacial detachment effects [48,49,50,51]. In addition, the peak values of all three models appear within a pull-off distance of approximately 1.5–2.0 Å, suggesting that the maximum resistance to separation occurs at the initial detachment stage, when van der Waals and electrostatic interactions between asphalt molecules and the ice surface are still strong. Overall, the simulated size dependence is broadly consistent with the macroscopic trend that larger interfaces tend to show lower nominal freeze-adhesion strength. However, the simulations should be interpreted as molecular-scale support for a size-dependent adhesion response rather than direct proof of the macroscopic defect-accumulation mechanism.

**Figure 8:**
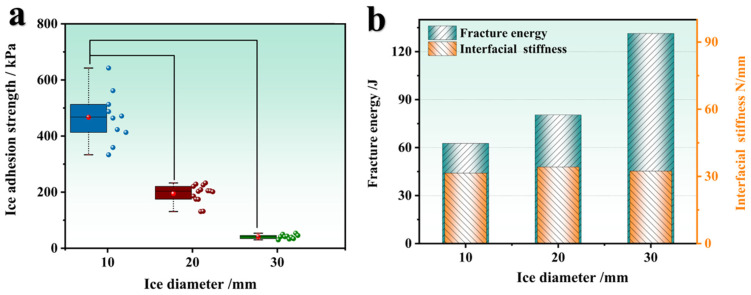
Results for different ice diameters: (**a**) ice–asphalt adhesion strength, coefficient of variation, and one-way ANOVA result showing a significant diameter effect on IAS (F(2,31) = 159.627, *p* < 0.001); (**b**) fracture energy and interfacial stiffness derived from the force–displacement curves.

The experimental trend shown in Figure 8 can be interpreted cautiously from two aspects. First, Figure 8a presents the nominal ice–asphalt adhesion strength (IAS) measured for different ice specimen diameters. As the interfacial diameter increases, the nominal contact area also increases, which may increase the probability of local weak regions, interfacial heterogeneity, uneven water distribution, and nonuniform load transfer at the ice–asphalt interface. As a result, larger interfaces are more likely to develop local weak spots, where debonding initiates first and then propagates along the interface. Thus, although larger specimens have a greater total contact area, their effective load-bearing capacity per unit area may decrease, leading to the observed reduction in nominal IAS. In contrast, the 10 mm specimens have a smaller nominal interface and may experience a relatively more uniform interfacial response, which is consistent with their higher nominal IAS.

Second, Figure 8b shows that fracture energy increases with increasing ice diameter, whereas interfacial stiffness remains within a relatively narrow range of 31.5–34.2 N/mm, with only a slight increase at 20 mm. This indicates that larger specimens require more total energy for complete separation, but it does not mean that the area-normalized adhesion resistance is enhanced. The reason is that larger-diameter specimens involve a larger total interfacial area and a longer crack propagation path, resulting in greater energy dissipation during debonding. At the same time, a larger interface may increase the probability of local weak regions, interfacial heterogeneity, and nonuniform load transfer, which is consistent with a lower peak response per unit area. Consequently, the seemingly contradictory trend of increasing fracture energy but decreasing nominal adhesion strength is physically reasonable. The relatively stable interfacial stiffness further suggests that the initial elastic response is governed more by the combined stiffness of the ice, asphalt layer, and loading system than by interfacial diameter alone.

The combined results of pull-off experiments and molecular dynamics simulations consistently indicate that model size affects interfacial adhesion strength during pull-off. Both the macroscopic experiments and microscopic simulations show the same overall trend, namely that smaller interfacial sizes correspond to higher nominal adhesion strength. This qualitative consistency supports a size-dependent interpretation of ice–asphalt pull-off behavior, while the different length scales and loading conditions of the two methods mean that the comparison should be treated as mechanistic correspondence rather than direct quantitative equivalence.

### 3.2. Effect of Temperature on Ice–Asphalt Interfacial Adhesion

**Figure 9:**
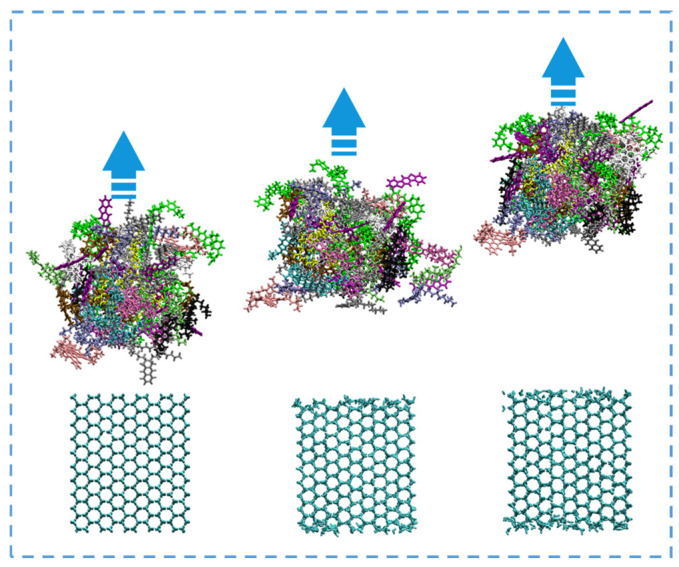
Snapshot of the asphalt–ice interfacial pull-off configuration at 220 K.

Pull-off simulations of the asphalt–ice interface were performed at six different temperatures, namely 220 K, 230 K, 240 K, 250 K, 260 K, and 273 K. The molecular dynamics snapshot of the asphalt–ice interfacial pull-off configuration at 220 K is shown in Figure 9.

**Figure 10:**
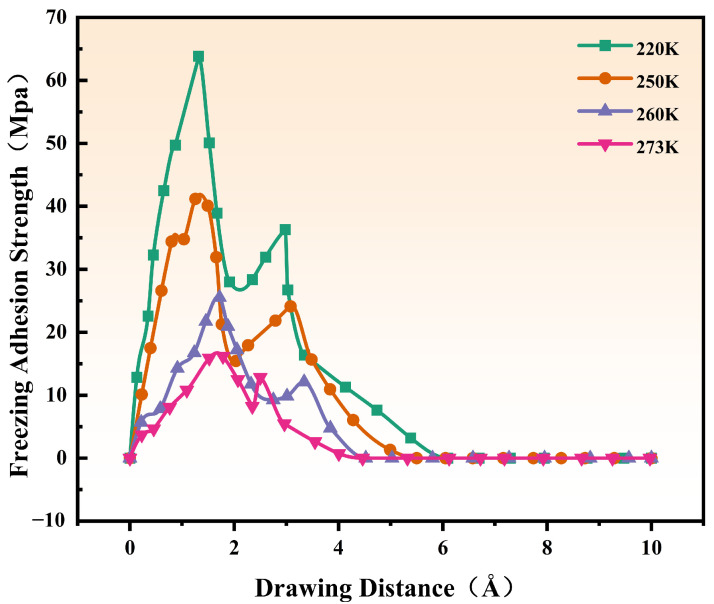
Adhesion strength as a function of pull-off distance for the asphalt–ice interfacial model at different temperatures.

The corresponding relationships between adhesion strength and pull-off distance at different temperatures are presented in Figure 10. As shown in the curves, the adhesion strength increases rapidly and reaches a peak at all temperatures. The highest adhesion strength is observed at 220 K, and the peak adhesion strength gradually decreases with increasing temperature. In addition, the pull-off distance corresponding to the peak value shows a slight increase as temperature rises. This difference in peak position reflects the rate at which intermolecular interactions are established during separation. At low temperatures, thermal motion is relatively weak, allowing intermolecular interactions at the interface to stabilize rapidly and reach their maximum strength at an earlier stage. In contrast, at higher temperatures, enhanced thermal motion disturbs the formation and stabilization of interfacial interactions, thereby delaying the occurrence of the peak and reducing the overall adhesion strength.

**Figure 11:**
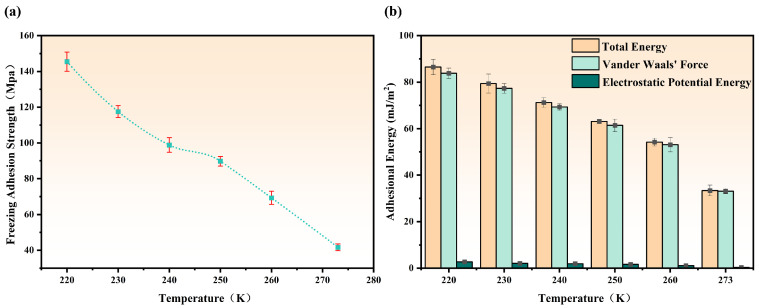
Temperature dependence of the interfacial adhesion behavior of the asphalt–ice model: (**a**) freezing adhesion strength at different temperatures; (**b**) short-range asphalt–ice interaction-energy descriptor and its LJ-SR and Coul-SR components during pull-off.

The short-range asphalt–ice interaction-energy descriptor was evaluated at six temperatures, and the total short-range term together with its Lennard–Jones/van der Waals and Coulombic components is presented in Figure 11b. With increasing temperature, the magnitude of the attractive short-range interaction decreases; the Lennard–Jones/van der Waals and Coulombic contributions show the same weakening tendency. This descriptor represents the combined short-range nonbonded contribution across the asphalt–ice interface, rather than the complete PME-decomposed interaction energy. Its gradual decrease with increasing temperature indicates that the interfacial interactions are stronger and more stable under lower-temperature conditions. Among these short-range components, the LJ-SR component is the major contribution to the asphalt–ice interaction-energy descriptor and decreases markedly with increasing temperature, suggesting that intensified thermal motion at elevated temperatures weakens short-range intermolecular attraction and reduces the effectiveness of interfacial contact. Although the electrostatic contribution is much smaller, it also decreases with increasing temperature, implying that thermal agitation disturbs the favorable relative orientation of interfacial molecules and slightly increases their average separation distance, thereby weakening electrostatic interactions. These energy results are consistent with the trend shown in Figure 11a, where the freezing adhesion strength decreases with increasing temperature and exhibits an approximately linear downward tendency. Therefore, the weakening of the short-range Lennard–Jones/van der Waals and Coulombic contributions provides molecular-scale support for the temperature-dependent decrease in asphalt–ice adhesion strength, but it should be interpreted as descriptor-level evidence rather than a direct quantitative conversion to macroscopic IAS.

**Figure 12:**
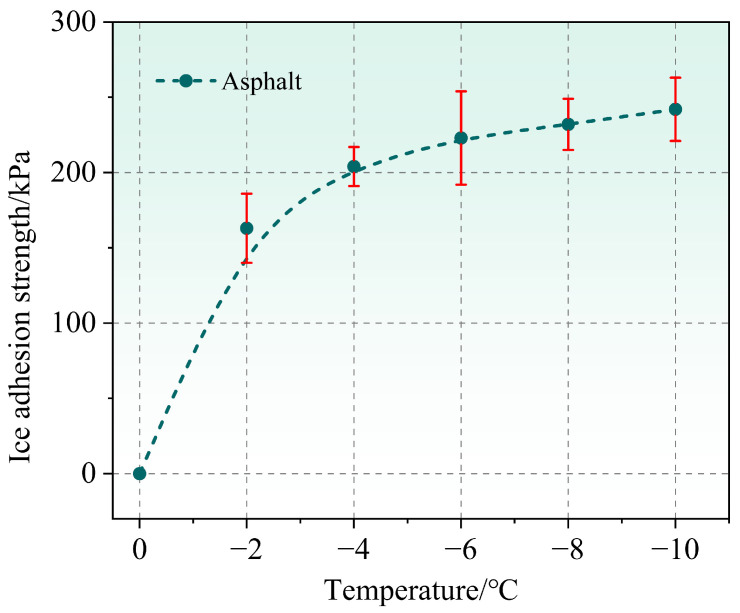
Temperature dependence of the pull-off adhesion strength at the ice–asphalt interface under low-temperature conditions (0 °C to −10 °C).

To compare the molecular simulation descriptors with laboratory-scale behavior, pull-off tests were performed to directly evaluate the interfacial adhesion force between the ice layer and the asphalt substrate, from which the corresponding adhesion strength was calculated. The experimental relationship between temperature and interfacial adhesion strength is shown in Figure 12. Within the low-temperature range from 0 °C to −10 °C, the adhesion strength increases markedly as temperature decreases. At 0 °C, a stable load-bearing ice–asphalt interface is difficult to establish, and the measured adhesion strength is therefore close to zero. As the temperature decreases further from −2 °C to −10 °C, the adhesion strength increases from 163 kPa to 242 kPa. It is also evident that the increase is relatively rapid in the range of 0 to −4 °C, whereas the growth rate becomes noticeably smaller from −4 to −10 °C, indicating that the interfacial adhesion behavior is more sensitive to temperature near the freezing point and becomes progressively less sensitive at lower temperatures.

This trend can be interpreted from the perspectives of intermolecular interaction and the physicochemical state of the ice surface. First, as temperature decreases, thermal fluctuations of interfacial molecules are reduced, which can help to maintain closer asphalt–ice contact and stronger short-range nonbonded interactions. The H-bond analysis indicates a low interfacial H-bond population; therefore, hydrogen bonding is treated here as an auxiliary local descriptor rather than the dominant cause of the temperature-dependent adhesion trend. As a result, the resistance of the interface to separation is enhanced. Second, a possible material-level contribution is the reduced mobility and time-dependent deformation capacity of the asphalt phase at lower temperature, which may limit load dissipation through internal deformation during pull-off. In addition, according to premelting theory, the quasi-liquid layer on the ice surface is relatively thick near the melting point and gradually becomes thinner as temperature decreases. A thinner quasi-liquid layer would be expected to reduce interfacial screening or lubrication and allow more effective asphalt–ice contact, which is consistent with stronger adhesion at lower temperatures. The slower increase in adhesion strength below −4 °C may indicate that, after interfacial contact and short-range interactions have become relatively stabilized, further cooling provides only limited additional enhancement. This interpretation remains qualitative because QLL thickness was not directly quantified in the present work.

### 3.3. Effect of Pull-Off Rate on Ice–Asphalt Interfacial Adhesion

**Figure 13:**
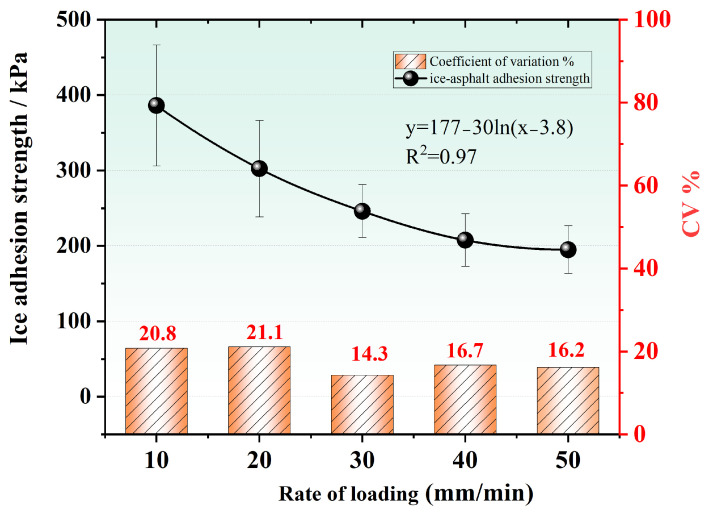
Effect of pull-off rate on asphalt–ice interfacial adhesion strength and coefficient of variation.

To investigate the effect of pull-off rate on the adhesion behavior of the asphalt–ice interface, pull-off tests were carried out at five different pull-off rates. The corresponding interfacial adhesion strength and coefficients of variation are presented in Figure 13. The IAS decreases progressively as the pull-off rate increases, indicating a clear rate-dependent pull-off response under the tested conditions. At lower pull-off rates, the interface may have more time for stress transfer and local interfacial adjustment, allowing the ice–asphalt contact region to accommodate the applied load more gradually. Possible time-dependent deformation of the asphalt phase may also contribute to this response. At higher pull-off rates, the shorter loading time may limit interfacial adjustment and possible time-dependent deformation, making the pull-off response more sensitive to local heterogeneity and weak interfacial regions. This interpretation is consistent with the lower nominal IAS observed at higher pull-off rates. Overall, the decrease in adhesion strength shows a nonlinear decaying tendency, with the rate effect becoming less pronounced at higher pull-off rates.

The error bars and coefficients of variation further show that the degree of data scatter remains at a comparable level for different pull-off rates, without a systematic increase or decrease as the pull-off rate increases, indicating that the repeatability and stability of the tests are generally acceptable. The relatively higher coefficient of variation at lower pull-off rates may be associated with the longer testing duration, during which the interfacial state is more sensitive to operational details and local heterogeneity. At higher pull-off rates, the shorter loading duration may reduce the cumulative influence of slow interfacial changes, but the more rapid debonding response can also make the measured peak force sensitive to local weak regions. Therefore, the variation in CV under different pull-off rates is interpreted as a descriptive measure of response variability, which may arise from local interfacial heterogeneity, rate-dependent debonding behavior, and possible time-dependent deformation of the asphalt layer.

Because the strain rates and pulling velocities accessible in molecular dynamics simulations are much higher than the experimental pull-off rates, five pull-off simulations were conducted at different strain rates to evaluate the effect of MD strain rate on the molecular-scale adhesion response of the asphalt–ice interface. The strain rates ranged from 2 × 10^9^ s^−1^ to 1 × 10^7^ s^−1^, corresponding to pulling velocities from 50 m/s to 0.1 m/s. In addition, a quasi-static tensile protocol was adopted, in which the system was equilibrated for 100 ps at each displacement step during pull-off. These MD rates are many orders of magnitude higher than the experimental pull-off rates; therefore, the MD rate analysis is used as a within-scale molecular sensitivity test rather than as a direct quantitative reproduction of the laboratory rate dependence.

**Figure 14:**
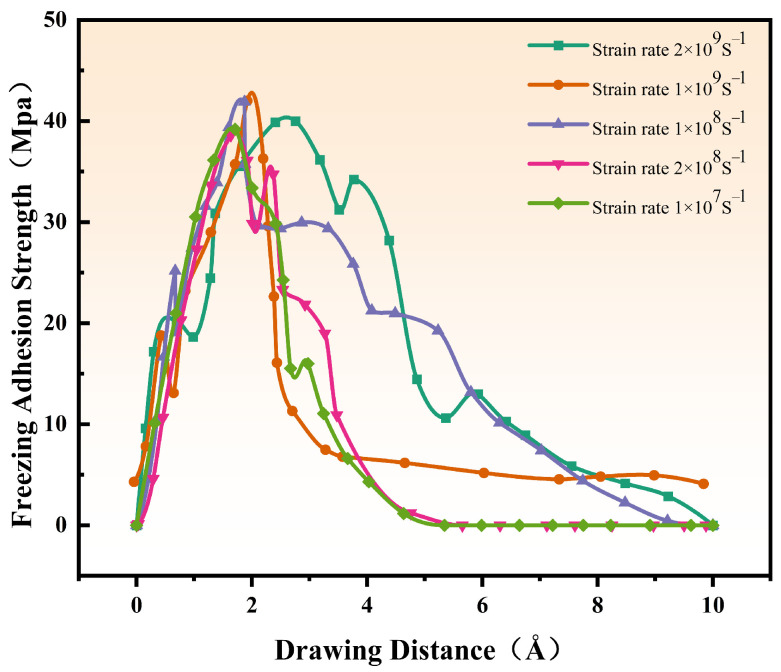
Adhesion strength as a function of pull-off distance for the asphalt–ice interfacial model at different strain rates.

The relationships between molecular-scale nominal adhesion strength and pull-off distance at different strain rates are shown in Figure 14. Although some differences can be observed in the peak magnitude, the peak position, and the post-peak decay behavior, the overall separation process remains similar for all strain rates. In general, the peak adhesion strengths are distributed within a relatively narrow range, and no clear monotonic dependence of peak adhesion strength on strain rate is observed. This suggests that, within the simulated range, the intrinsic interfacial adhesion of the asphalt–ice system is not highly sensitive to strain rate. However, the strain rate does influence the detailed evolution of the detachment process. At some higher strain rates, the peak appears at a slightly larger pull-off distance and the post-peak response becomes more extended, indicating that interfacial stress redistribution and molecular rearrangement are affected by the available relaxation time during pull-off.

The weak strain-rate dependence observed in the simulations may be attributed to the idealized nature of the molecular model and the quasi-static loading protocol. In atomistic simulations, the interface is structurally uniform and does not fully account for microscale defects, pores, or local stress concentrators that are often present in macroscopic experiments. Moreover, the 100 ps equilibration applied at each displacement step allows partial structural relaxation, which further reduces the apparent rate sensitivity of the interfacial response. Therefore, compared with macroscopic tests, the strain-rate effect in molecular dynamics simulations mainly reflects intrinsic molecular interaction and local relaxation behavior, rather than defect-amplified failure processes. The MD simulations quantify a high-rate molecular response of an idealized asphalt–ice interface. The two datasets are therefore compared as scale-aware mechanistic correspondence, not as directly convertible rate laws.

### 3.4. Single-Molecule Adhesion Behavior of Representative Asphalt Molecules on Ice

**Figure 15:**
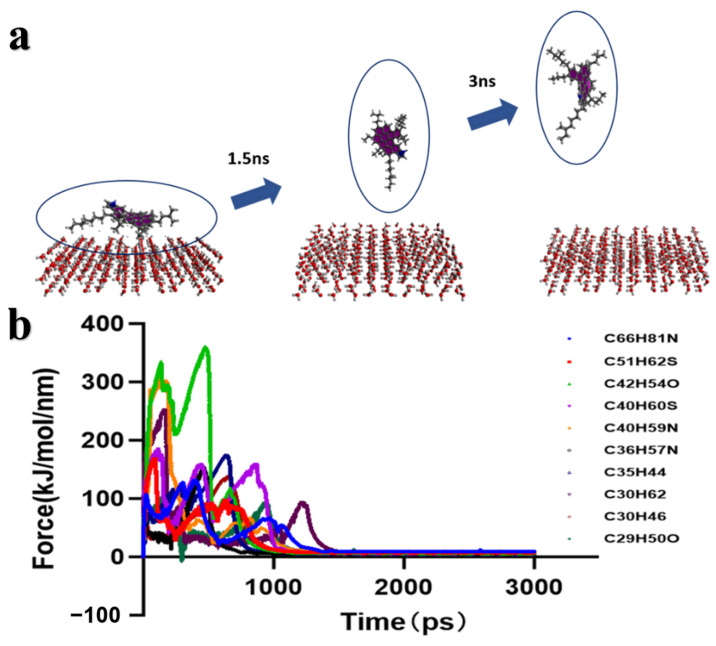
(**a**) Dynamic snapshots of the simulated pull-off process of the representative asphaltene molecule C66H81N from the ice surface; (**b**) force–time curves of representative asphalt molecules during pull-off from the ice surface.

As shown in Figure 15a, taking the asphaltene-like representative molecule C66H81N as an example, its spatial position and molecular configuration evolve continuously during pull-off. During progressive interfacial separation, the nitrogen-containing polar site remains relatively close to the ice surface, indicating directional polar interactions between this site and the ice substrate. This behavior may be associated with the polarity of the nitrogen-containing functional group, which may participate in directional polar interactions and possible local hydrogen bonding with interfacial ice/water molecules. As a result, the molecule retains a certain interfacial anchoring tendency during the early stage of detachment. In addition, the large conjugated framework and relatively planar local structure of the asphaltene molecule may also contribute to stabilizing its contact with the ice surface. Therefore, the dynamic pull-off snapshots suggest that both polar heteroatom sites and molecular configuration are associated with interfacial retention during detachment. To further quantify the differences in interfacial interactions among molecules, the mechanical responses of different molecules against the ice surface were then analyzed.

The force–time curves of representative asphalt molecules during pull-off are shown in Figure 15b. Clear differences are observed among molecules in peak force, interaction duration, and decay behavior, indicating the strong component dependence of interfacial adhesion. After grouping the molecules into asphaltenes, resins, aromatics, and saturates, it is evident that asphaltenes generally exhibit the highest peak forces and the longest effective interaction durations, suggesting the strongest interactions with the ice surface. Polar aromatic molecules reach their peak forces rapidly but decay quickly during pull-off, indicating strong but short-range and direction-sensitive interactions. Resin molecules show intermediate interaction strength with a smoother decay profile, whereas saturates exhibit the weakest forces because of the absence of significant polar sites. Overall, the interfacial adhesion capability of asphalt molecules is closely related to polar heteroatom content, molecular planarity, and aromatic conjugation.

**Figure 16:**
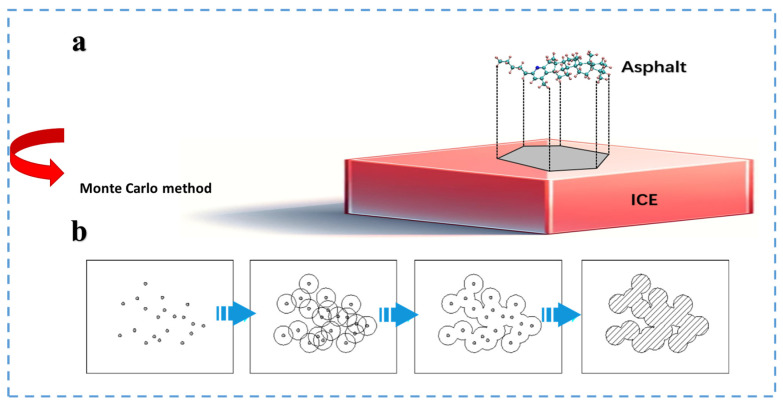
Determination of the projected contact area of a single asphalt molecule on the ice surface: (**a**) schematic illustration of the molecular projection area used to define the contact area; (**b**) calculation procedure of the projected area based on the Monte Carlo method.

To derive adhesion force and adhesion strength from the force–time and displacement–time curves, the maximum pull-off force was taken as the characteristic molecular adhesion force. To further determine the adhesion strength of a single molecule on the ice surface, the contact area between the molecule and the ice layer must be defined. Because the representative asphalt molecules differ in molecular size and spatial conformation, their effective contact areas with the ice surface are not identical. In this study, the projected area of each asphalt molecule approaching the ice surface was defined as its contact area. A typical three-dimensional molecular configuration and its corresponding planar projection area are illustrated in Figure 16a. Therefore, the adhesion strength of each molecule is jointly determined by its adhesion force and projected contact area.

To obtain the molecular-scale nominal adhesion strength of each representative asphalt molecule, the maximum pull-off force was normalized by the Monte Carlo projected molecular area. The projected area was estimated using a point-in-projection procedure, as illustrated in Figure 16b, with an effective atomic projection radius of 2.0 Å used as the baseline. Changing the projection radius altered the absolute projected area and the resulting area-normalized molecular stress, as expected, but the relative ranking of the 12 representative asphalt molecules remained stable. Using the 2.0 Å result as the baseline, the Spearman rank correlation was 1.0 for both the 1.8 and 2.2 Å calculations. Therefore, the projected-area normalization is used as a relative molecular descriptor rather than as a uniquely defined physical contact area.

**Figure 17:**
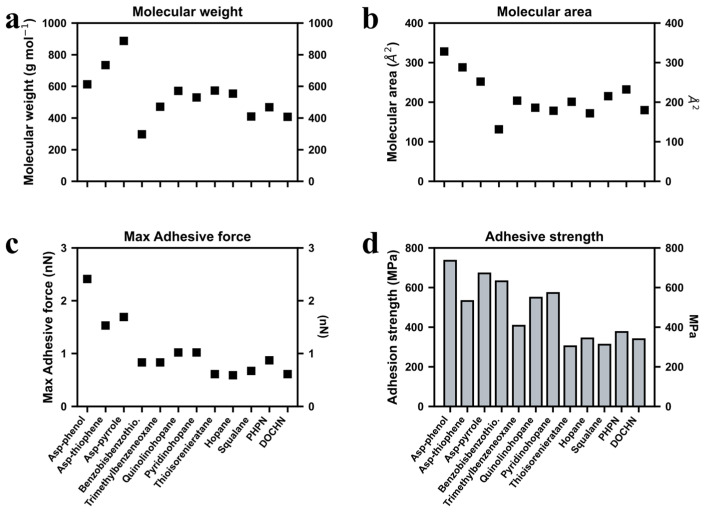
Comparison of the molecular descriptors and pull-off parameters of 12 representative asphalt molecules on the ice surface: (**a**) molecular weight, (**b**) projected molecular area, (**c**) maximum pull-off force, and (**d**) molecular-scale nominal adhesion strength.

The maximum single-molecule pull-off force of each molecule was obtained from the pull-off force curve, and the corresponding molecular-scale nominal adhesion strength was obtained by normalizing this force with the Monte Carlo projected molecular area. The molecular weight, projected molecular area, maximum pull-off force, and molecular-scale nominal adhesion strength of the 12 representative asphalt molecules are summarized in Figure 17, and the detailed values are listed in Table 3. The unit conversion and projected-area normalization are described in the Supplementary Information, and these MPa-scale values are used as relative molecular descriptors rather than direct numerical predictions of the experimental kPa-scale IAS.

The 12 representative asphalt molecules exhibit clear differences in molecular mass, projected molecular area, maximum pull-off force, and molecular-scale nominal adhesion strength, indicating the presence of composition-dependent single-molecule adhesion descriptors at the asphalt–ice interface. In general, asphaltene species and several polar/aromatic molecules exhibit relatively high maximum pull-off forces and molecular-scale nominal adhesion strengths, whereas those for saturate-like molecules tend to be lower. A comparison of Figure 17b–d further suggests that molecular-scale nominal adhesion strength is not related to molecular mass alone, but reflects the combined influence of maximum pull-off force and projected molecular area. Molecules with relatively planar aromatic frameworks and dense atomic distributions can establish more effective contact with the ice surface, thereby enhancing intermolecular attraction. In addition, heteroatoms such as N, O, and S introduce localized polar sites, which can contribute to electrostatic interactions, dipole-related interactions, and potential hydrogen-bonding effects at the interface. As a result, asphaltenes and certain polar aromatics generally exhibit stronger adhesion than saturates and weakly polar species. Overall, molecular planarity, aromatic conjugation, heteroatom-induced polarity, and projected molecular area are important descriptors associated with the single-molecule adhesion response on ice, rather than exclusive factors that fully determine adhesion.

**Figure 18:**
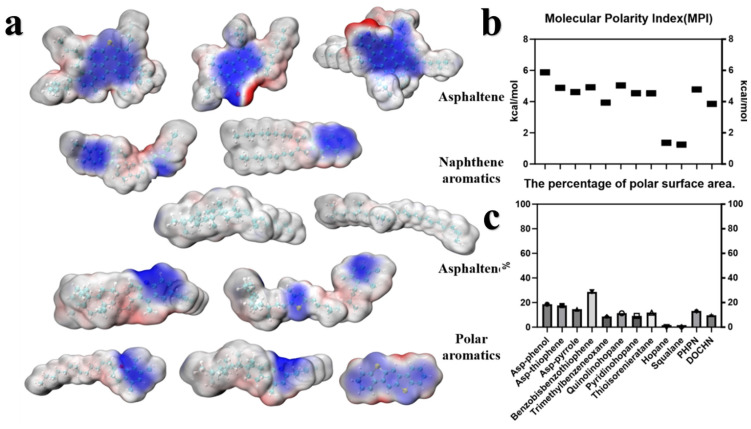
Polarity characterization of the 12 representative asphalt molecules: (**a**) molecular surface electrostatic potential distributions; (**b**) molecular polarity index (MPI); and (**c**) fraction of polar surface area.

As shown in Figure 18a, the 12 representative asphalt molecules exhibit distinct surface electrostatic potential distributions. In general, asphaltenes and some polar aromatic molecules display more pronounced positive and negative potential regions, as reflected by the larger and more intense red/blue areas on the molecular surface. This indicates a stronger non-uniformity of charge distribution and hence a higher degree of molecular polarity. In contrast, saturate molecules exhibit relatively uniform electrostatic potential distributions with fewer and weaker polar regions, suggesting much lower surface polarity. These differences in electrostatic potential distribution provide a direct electrostatic basis for the variation in interfacial adhesion behavior among different asphalt components.

The molecular polarity index (MPI) values of the 12 representative molecules are further summarized in Figure 18b. Asphaltenes generally show relatively high MPI values, while some polar aromatic molecules also exhibit elevated MPI values compared with weakly polar or nonpolar species. By contrast, saturates show the lowest MPI values. This indicates that aromatic molecules containing heteroatoms such as N, O, and S usually possess stronger charge-distribution asymmetry and thus higher molecular polarity. When compared with the molecular-scale nominal adhesion strengths, the MPI values show a moderate positive association with single-molecule adhesion response, with Pearson’s r = 0.691 and R^2^ = 0.477. This suggests that molecular polarity is an important descriptor associated with molecular-level asphalt–ice adhesion, rather than the only factor determining adhesion.

As shown in Figure 18c, the fraction of polar surface area also varies markedly among the representative molecules. In general, molecules with a larger proportion of polar surface area tend to show higher molecular-scale nominal adhesion strength, because a larger exposed polar region may increase the opportunity for contact between polar sites and the ice surface during pull-off. This can contribute to Coulombic, dipole-related, and possible local hydrogen-bonding interactions. Nevertheless, adhesion capability is not determined by polar-area fraction alone, but is also influenced by the spatial distribution of polar regions, molecular planarity, and contact configuration. Therefore, the combined results of Figure 18a–c demonstrate that surface electrostatic potential distribution, MPI, and polar surface area fraction consistently characterize molecular polarity from different perspectives, and that stronger molecular polarity is generally associated with higher single-molecule adhesion at the ice–asphalt interface.

## 4. Conclusions

This study combined controlled pull-off tests with representative molecular dynamics simulations to investigate asphalt–ice adhesion in an asphalt–ice interfacial system. The main conclusions are as follows:

1. Different representative asphalt molecules exhibit different adhesion strengths during pull-off from the ice surface. Asphaltene species and several polar/aromatic molecules generally show higher molecular-scale nominal adhesion strengths than saturate-like molecules, which is associated with molecular planarity, aromatic framework, projected molecular area, and heteroatom-induced polarity. This polarity-related trend is consistent with the MPI analysis, indicating that molecular polarity is an important descriptor of molecular-level asphalt–ice adhesion rather than the only controlling factor.
2. The adhesion strength of the asphalt–ice interface is closely related to temperature. The pull-off tests show that IAS increases from approximately 163 to 242 kPa as temperature decreases from −2 to −10 °C, and the molecular simulations provide descriptor-level support for stronger asphalt–ice interactions at lower temperature. With increasing temperature, the short-range asphalt–ice interaction-energy descriptor becomes less negative, and the short-range Lennard–Jones/van der Waals and Coulombic contributions both weaken.
3. The adhesion strength of the asphalt–ice interface is also related to interfacial size. The pull-off tests show that nominal IAS decreases as ice diameter increases, whereas the total fracture energy increases because larger specimens involve a larger contact area and greater energy dissipation during separation. Molecular simulations provide qualitatively consistent descriptor-level support, with smaller interfacial models showing higher-molecular-scale nominal adhesion responses.
4. Pull-off rate affects the macroscopic ice–asphalt adhesion response. Experimentally, IAS decreases as the pull-off rate increases under the tested laboratory conditions. In the high-rate MD simulations, changes in strain rate mainly affect the molecular detachment pathway, while the peak molecular-scale nominal adhesion response does not show the same monotonic rate dependence as the laboratory IAS. Thus, the MD rate analysis is treated as a within-scale molecular sensitivity test rather than a directly convertible rate law.

## Figures and Tables

**Table 1:** Molecular composition and fundamental information of the representative AAA-1 asphalt model used for molecular simulations [19].

Molecule	Molecular Formula	Number in Model System	Atom Mass Fraction (C_arom_)	Atom Mass Fraction (S)	Atom Mass Fraction (N)
Asphaltene-thiophene	C_51_H_62_S	3	0.42	0.000	0.000
Asphaltene-pyrrole	C_66_H_81_N	2	0.41	0.000	0.16
Asphaltene-phenol	C_42_H_50_O	3	0.41	0.045	0.000
Squalane	C_30_H_62_	4	0.00	0.000	0.000
Hopane	C_29_H_50_	4	0.00	0.000	0.000
PHPN	C_35_H_44_	11	0.41	0.000	0.000
DOCHN	C_30_H_46_	13	0.30	0.000	0.000
Quinolinohopane	C_34_H_47_N	4	0.20	0.000	0.025
Thioisorenieratane	C_40_H_60_S	4	0.34	0.056	0.000
Trimethylbenzeneoxane	C_29_H_50_O	5	0.17	0.000	0.000
Pyridinohopane	C_30_H_45_N	4	0.12	0.000	0.028
Benzobisbenzothiophene	C_18_H_10_S_2_	15	0.74	0.22	0.000

**Table 2:** Basic parameters of the TIP4P-ICE water model [37].

	ε/k (K)	σ (Å)	q_(H) (e)_	q_(O) (e)_	q_(M) (e)_	r_OM (Å)_	r_OH (Å)_	∠HOH (°)
TIP4P-ICE	106.1	3.1668	0.5897	0	−1.1794	0.15	0.9572	104.52

**Table 3:** Projected molecular area, molecular weight, maximum pull-off force, and molecular-scale nominal adhesion strength of representative asphalt molecules on ice.

Molecule	Projected Area (Å^2^)	Molecular Weight	Maximum Pull-Off Force (nN)	Molecular-Scale Nominal Adhesion Strength (MPa)
Asphaltene-thiophene	287.95	734.0	1.53	531.3
Asphaltene-pyrrole	251.81	887.0	1.69	671.1
Asphaltene-phenol	328.06	613.0	2.41	734.6
Squalane	214.87	409.0	0.67	311.8
Hopane	171.84	554.0	0.59	343.3
PHPN	231.93	468.0	0.87	375.1
DOCHN	180.01	407.0	0.61	338.9
Quinolinohopane	185.78	571.0	1.02	549.0
Thioisorenieratane	200.87	573.0	0.61	303.7
Trimethylbenzeneoxane	203.85	471.0	0.83	407.2
Pyridinohopane	178.32	530.0	1.02	572.0
Benzobisbenzothiophene	131.45	297.0	0.83	631.4

## Data Availability

The original contributions presented in this study are included in the article/Supplementary Material. Further inquiries can be directed to the corresponding author.

## Supplementary Materials

The following supporting information can be downloaded at https://www.mdpi.com/article/10.3390/ma19132929/s1, Figure S1a. Bulk asphalt 5 ns NPT equilibration with Parrinello–Rahman pressure coupling, including potential energy, temperature, pressure, and density. The 0–1 ns segment is treated as the initial segment, and summary statistics are calculated from the 1–5 ns analysis window. Figure S1b. Asphalt-ice interface equilibration metrics across 220, 230, 240, 250, 260, and 273 K over 5 ns. The 0–1 ns segment is treated as the initial segment, and the 1–5 ns window is used for quantitative summaries. Figure S1c. Asphalt-ice interface geometry stability across 220, 230, 240, 250, 260, and 273 K over 5 ns, including COM z-separation, minimum asphalt-ice distance, and contact count within 0.35 nm. Figure S2a. Mean interfacial hydrogen-bond count across temperatures for the asphalt-ice interface, calculated from the 1–5 ns analysis window. Error bars represent block SEM from 500 ps time blocks. Figure S3. Short-range asphalt-ice interaction energy (Coul-SR + LJ-SR) across temperatures, recalculated from 5 ns trajectories. Error bars represent block SEM from the 1–5 ns analysis window and are not independent repeat-simulation error bars. Figure S4. Exploratory association between the surface-electrostatic-potential molecular polarity index and molecular-scale nominal adhesion strength. The descriptor-level regression is reported as an association, not as a single-variable causal law.
