# Supplementary material for: Multiscale Investigation of the Factors Governing Ice–Asphalt Interfacial Adhesion Strength: Insights from Pull-Off Tests and Molecular Simulations
Source: Materials (Basel). 2026 Jul 7;19(13):2929. doi: 10.3390/ma19132929 (PMC13362902; doi:10.3390/ma19132929)
Supplement: Supplementary file 1 [file materials-19-02929-s001.zip › materials-4370908-supplementary.pdf]

Supporting Information

Multiscale Investigation of the Factors Governing Ice–Asphalt Interfacial Adhesion Strength: Insights from Pull-Off Tests and Molecular Simulations

Teng Yuan[1,2,3], Yunhao Jiao[1], Qian Su[4], Yujin Yao[1], Huaxin Chen[1], Yongchang Wu[1,]*

1 School of Materials Science and Engineering, Chang'an University, Xi'an 710064, China

2 Xinjiang Transport Planning Survey and Design Institute Co., Ltd., Urumqi 830006, China

3 Xinjiang Key Laboratory for Safety and Health of Transportation Infrastructure in Alpine and High-altitude Mountainous Areas, Urumqi 830006, China

4 The Engineering Design Academy of Chang'an University Co., Ltd., Xi'an 710064, China

* Correspondence: ywu@chd.edu.cn (Y. Wu)

Keywords: asphalt; ice adhesion; molecular dynamics simulation; interfacial interaction

Supplementary Information

2.1. Supplementary details of molecular dynamics simulations.

2.1.1. Molecular model and force-field settings

The asphalt phase was represented by the literature-established 12-component AAA-1 model. This model was used as a representative multicomponent asphalt proxy, not as a molecule-by-molecule reconstruction of a specific experimental binder. The ice phase was described with the TIP4P/Ice water model, and the simulations were performed using

GROMACS 2020.3.

The asphalt molecules were described using the OPLS-AA framework. Long-range electrostatic interactions were treated with particle mesh Ewald (PME), and short-range interactions were evaluated using a cutoff scheme. NPT denotes a constant number of particles, pressure, and temperature.

In the force-field description, the total potential energy was expressed as the sum of bonded and nonbonded contributions:

$$E_{tot} = E_{bond} + E_{angle} + E_{dihedral} + E_{nonbonded} \tag{S1}$$

$$E_{bond} = \sum_{bonds} k_r (r - r_0)^2 \tag{S2}$$

$$E_{angle} = \sum_{angles} k_\theta (\theta - \theta_0)^2 \tag{S3}$$

$$E_{dihedral} = \sum_{dihedrals} \sum_{n=1}^{4} \frac{V_n}{2} [1 + cos(n\varphi - \varphi_n)] \tag{S4}$$

$$E_{nonbonded} = \sum_{i>j} [4\varepsilon_{ij}((\frac{\sigma_{ij}}{r_{ij}})^{12} - (\frac{\sigma_{ij}}{r_{ij}})^{6}) + \frac{q_i q_j}{4\pi\varepsilon_0 r_{ij}}] \tag{S5}$$

Here, r, θ, and φ denote bond length, bond angle, and dihedral angle, respectively. The terms q_i and q_j are atomic charges, r_{ij} is the interatomic distance, and ε_0 is the vacuum permittivity.

The Lennard-Jones cross-interaction parameters followed the combination rule used in the simulation topology (comb-rule = 2). Therefore, σ_{ij} is obtained from the arithmetic mean of σ_i and σ_j, whereas ε_{ij} is obtained from the geometric mean of ε_i and ε_j:

$$\sigma_{ij} = \frac{\sigma_i + \sigma_j}{2} \tag{S6}$$

$$\varepsilon_{ij} = \sqrt{\varepsilon_i \varepsilon_j} \tag{S7}$$

2.1.2. Five-nanosecond stability and equilibration validation

Additional 5 ns validation trajectories were analyzed to evaluate the stability of the bulk

asphalt and asphalt-ice interface models. For these trajectories, the 0-1 ns segment was treated as the initial segment, and quantitative summaries were calculated from the 1-5 ns analysis window. Supplementary Figures S1a–S1c provide the corresponding time-series and geometry-stability evidence.

2.1.3. Interfacial hydrogen-bond analysis

Interfacial hydrogen bonds between asphalt and ice were analyzed as a supplementary local descriptor. The geometric criterion was:

$$r_{DA} \leq 0.35\,nm \text{ and } \angle D - H \cdots A \geq 150° \quad \text{(S8)}$$

where r_{DA} is the donor-acceptor distance and ∠D-H···A is the donor-hydrogen-acceptor angle. The analysis used the 1-5 ns window of the 5 ns trajectories. Hydrogen bonding is interpreted as a local interfacial descriptor and is not treated as the dominant cause of the temperature-dependent adhesion trend.

2.1.4. Short-range asphalt-ice interaction energy and block uncertainty

The supplementary interaction-energy descriptor was defined as the short-range asphalt-ice interaction energy. It was calculated as Coul-SR + LJ-SR from rerun analysis:

$$E_{int,SR} = E_{Coul-SR} + E_{LJ-SR} \quad \text{(S9)}$$

This descriptor is limited to short-range pair terms and should not be interpreted as a complete PME-decomposed interaction energy. Error bars were estimated as block standard errors from 500 ps blocks in the 1–5 ns analysis window; they are not independent repeat-simulation error bars.

2.1.5. Single-molecule nominal adhesion-strength conversion

The nominal single-molecule adhesion strength was calculated from the maximum pull-off force and the Monte Carlo projected molecular area:

$$\sigma_{mol}\ (\text{MPa}) = \frac{F_{max}\ (\text{nN})}{A_{proj}\ (\text{Å}^2)} \times 10^5 \tag{S10}$$

$$1\ \text{nN Å}^{-2} = 10^{11}\ \text{Pa} = 10^5\ \text{MPa} \tag{S11}$$

These values are molecular-scale nominal stresses and should not be directly compared with experimental kPa-scale IAS values.

2.1.6. Monte Carlo projected molecular area and radius sensitivity

The projected molecular area was estimated using a Monte Carlo point-in-projection procedure with 250,000 random points in the two-dimensional molecular bounding rectangle:

$$A_{proj} = A_{box} \cdot \frac{N_{in}}{N_{total}} \tag{S12}$$

Atomic projection radii of 1.8, 2.0, and 2.2 Å were evaluated, with 2.0 Å used as the baseline. Changing the projection radius altered the absolute projected molecular area and the resulting molecular-scale nominal stress, but the relative ranking of the 12 representative asphalt molecules remained unchanged. Using the 2.0 Å result as the baseline, the Spearman rank correlation was 1.0 for both the 1.8 and 2.2 Å calculations. Therefore, the projected-area normalization is used as a relative molecular descriptor rather than as a uniquely defined physical contact area.

2.1.7. Molecular polarity index and MPI-adhesion regression

The molecular polarity index (MPI) was used as a surface electrostatic-potential descriptor for the representative asphalt molecules:

$$\text{MPI} = \frac{1}{S}\int_S |V(r)|dS \tag{S13}$$

where S is the molecular surface and V(r) is the electrostatic potential at surface point r. The exploratory regression between MPI and molecular-scale nominal adhesion strength was expressed as:

$$\sigma_{mol} = \beta_0 + \beta_1 \text{MPI} + \varepsilon \tag{S14}$$

The regression included 12 molecules and gave Pearson's r = 0.691 and R-squared = 0.477. This result is interpreted as a moderate positive association rather than evidence that MPI alone determines adhesion.

2.2. Supplementary details of experimental pull-off tests

This section reports additional experimental details for the asphalt–ice pull-off tests described in Section 2.2 of the main manuscript, including the IAS calculation, standard test condition, rate-effect condition, replicate handling, and apparatus-validation boundary.

2.2.1. IAS calculation and standard pull-off protocol

Asphalt–ice adhesion strength (IAS) was calculated from the maximum pull-off force and the nominal asphalt–ice contact area:

$$\text{IAS} = \frac{F_{max}}{A_c} \tag{S15}$$

$$A_c = \frac{\pi d^2}{4} \tag{S16}$$

where F_{max} is the maximum pull-off force, A_c is the nominal contact area, and d is the ice-column diameter. Unless otherwise specified, the standard pull-off tests used a 20 mm ice column, corresponding to A_c = 3.14 cm^2, and a pull-off rate of 40 mm/min. Each reported IAS value for the asphalt samples was averaged from at least 10 pull-off measurements.

When descriptive repeatability parameters were reported, they were calculated from the replicate IAS values as follows:

$$\overline{x} = \frac{1}{n}\sum_{i=1}^{n} x_i \tag{S17}$$

$$s = \sqrt{\frac{\sum_{i=1}^{n}(x_i - \overline{x})^2}{(n - 1)}} \tag{S18}$$

$$\mathrm{CV} = \frac{100s}{\bar{x}} \tag{S19}$$

Here, xi is an individual IAS measurement, x̄ is the mean IAS, s is the sample standard deviation, and CV is the coefficient of variation. The Supplementary Information reports descriptive repeatability metrics only.

For the rate-effect experiment, the pull-off rate was varied from 10 to 50 mm/min and is treated separately from the standard 40 mm/min condition. Before pull-off testing, the asphalt-coated substrates and ice moulds were cleaned, pre-cooled, frozen, and thermally equilibrated following the procedure described in the main manuscript.

2.2.2. Apparatus validation and experimental parameter summary

The asphalt–ice adhesion tests were performed using a self-developed integrated testing system. The system included environmental-control, force-measurement, software-control, and test-platform subsystems. The apparatus reliability is described in the main manuscript through PDMS-based calibration, parameter-sensitivity evaluation, and comparison with literature-reported experimental data.

Supplementary figures

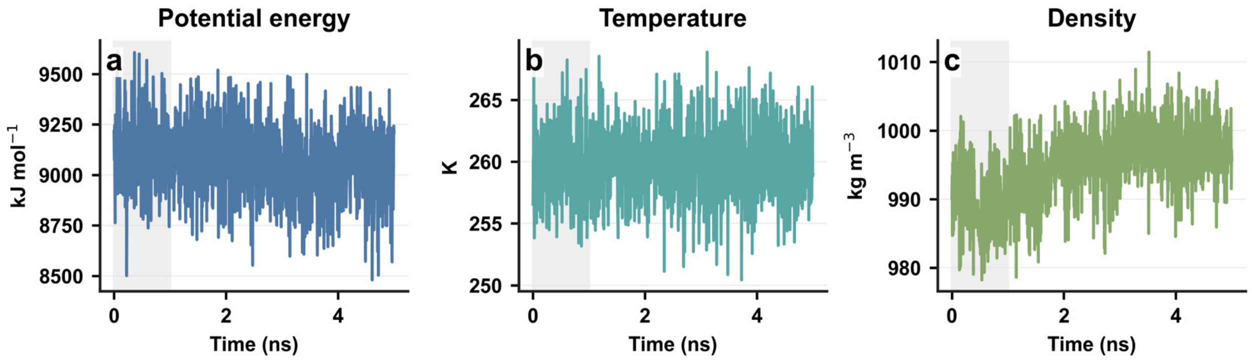

Figure S1a. Bulk asphalt 5 ns NPT equilibration with Parrinello–Rahman pressure coupling, including potential energy, temperature, pressure, and density. The 0-1 ns segment is treated

as the initial segment, and summary statistics are calculated from the 1-5 ns analysis window.

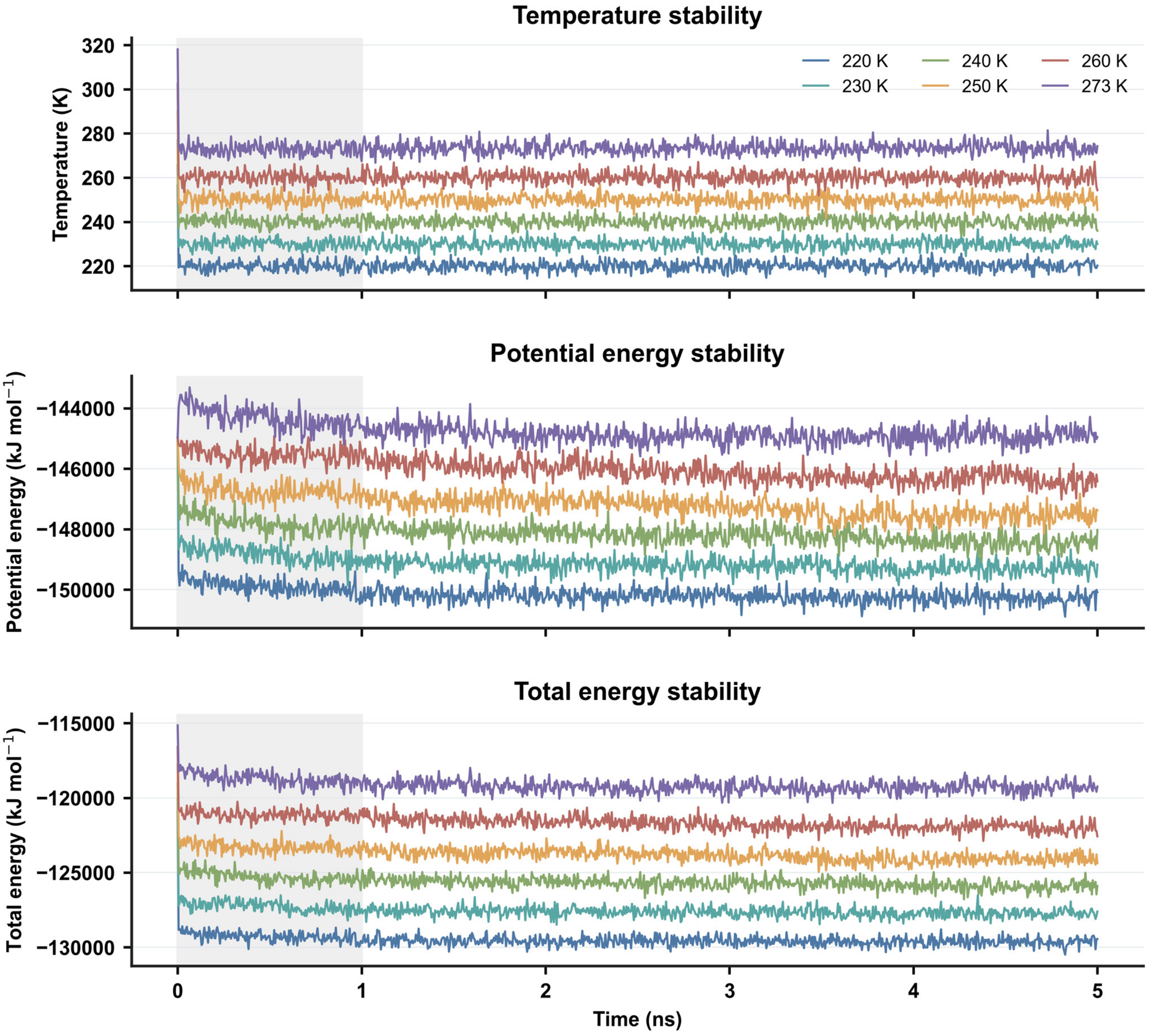

Figure S1b. Asphalt-ice interface equilibration metrics across 220, 230, 240, 250, 260, and 273 K over 5 ns. The 0-1 ns segment is treated as the initial segment, and the 1-5 ns window is used for quantitative summaries.

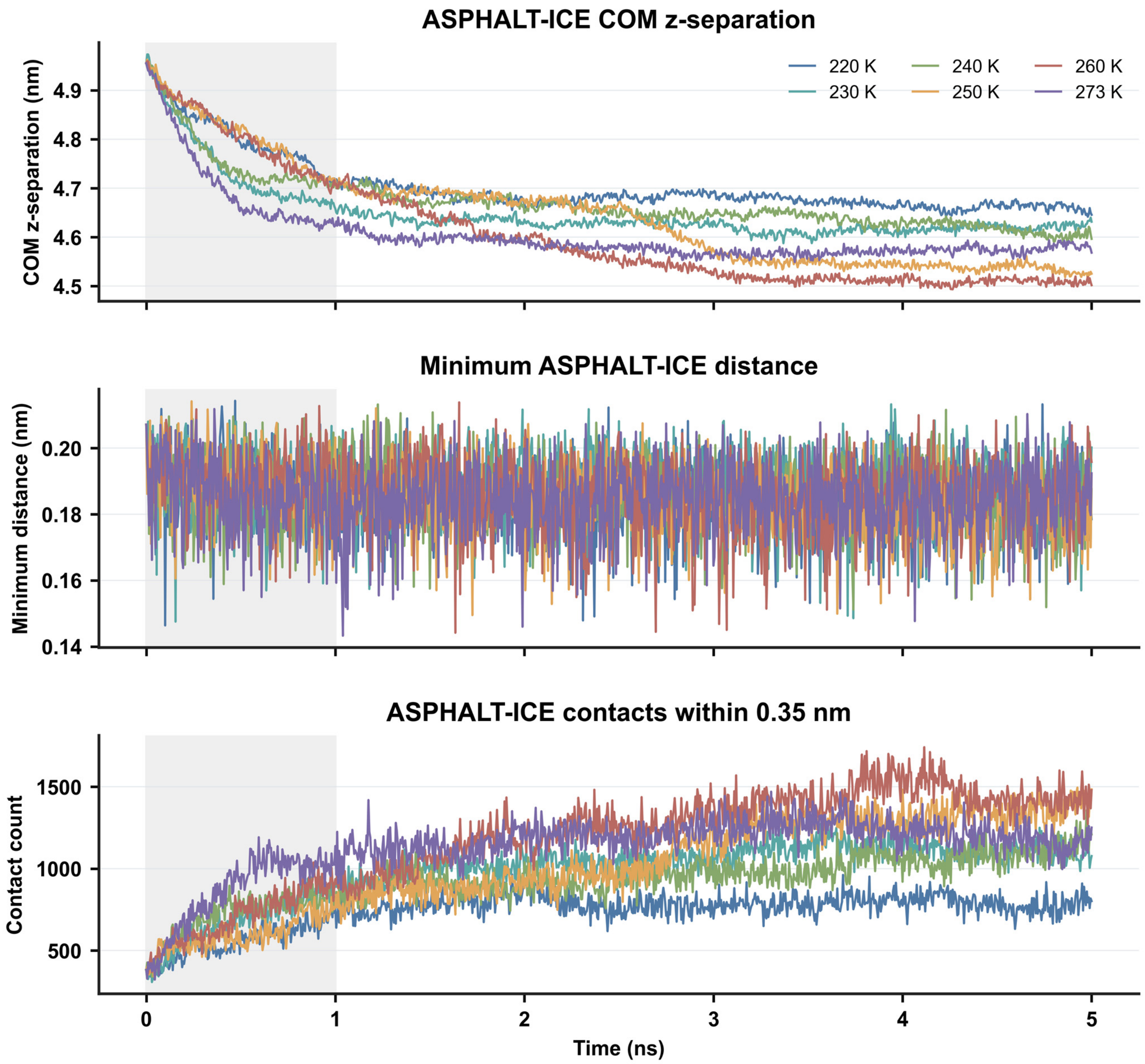

Figure S1c. Asphalt-ice interface geometry stability across 220, 230, 240, 250, 260, and 273 K over 5 ns, including COM z-separation, minimum asphalt-ice distance, and contact count within 0.35 nm.

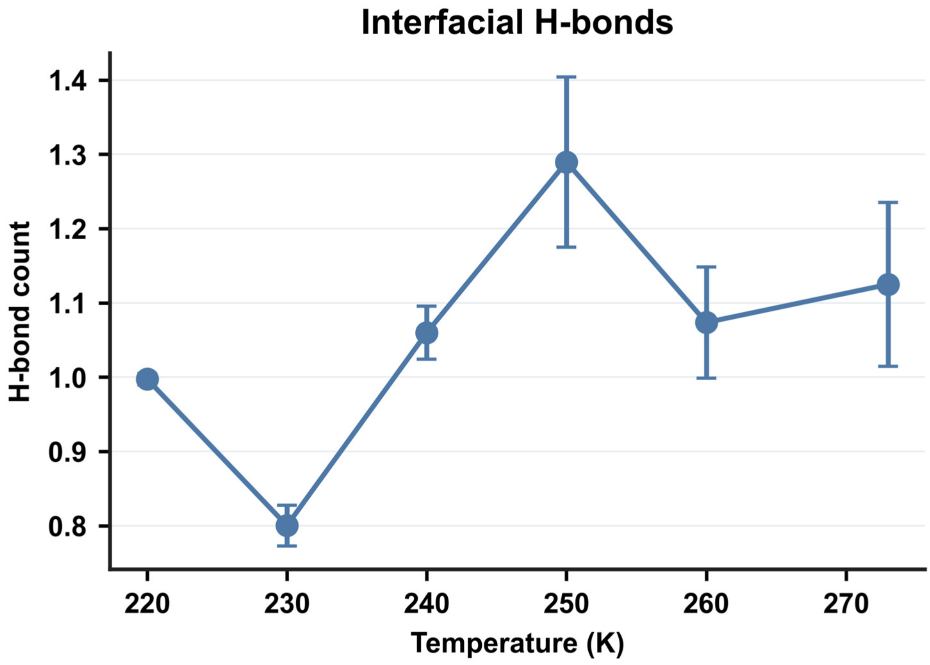

Figure S2. Mean interfacial hydrogen-bond count across temperatures for the asphalt-ice interface, calculated from the 1-5 ns analysis window. Error bars represent block SEM from 500 ps time blocks.

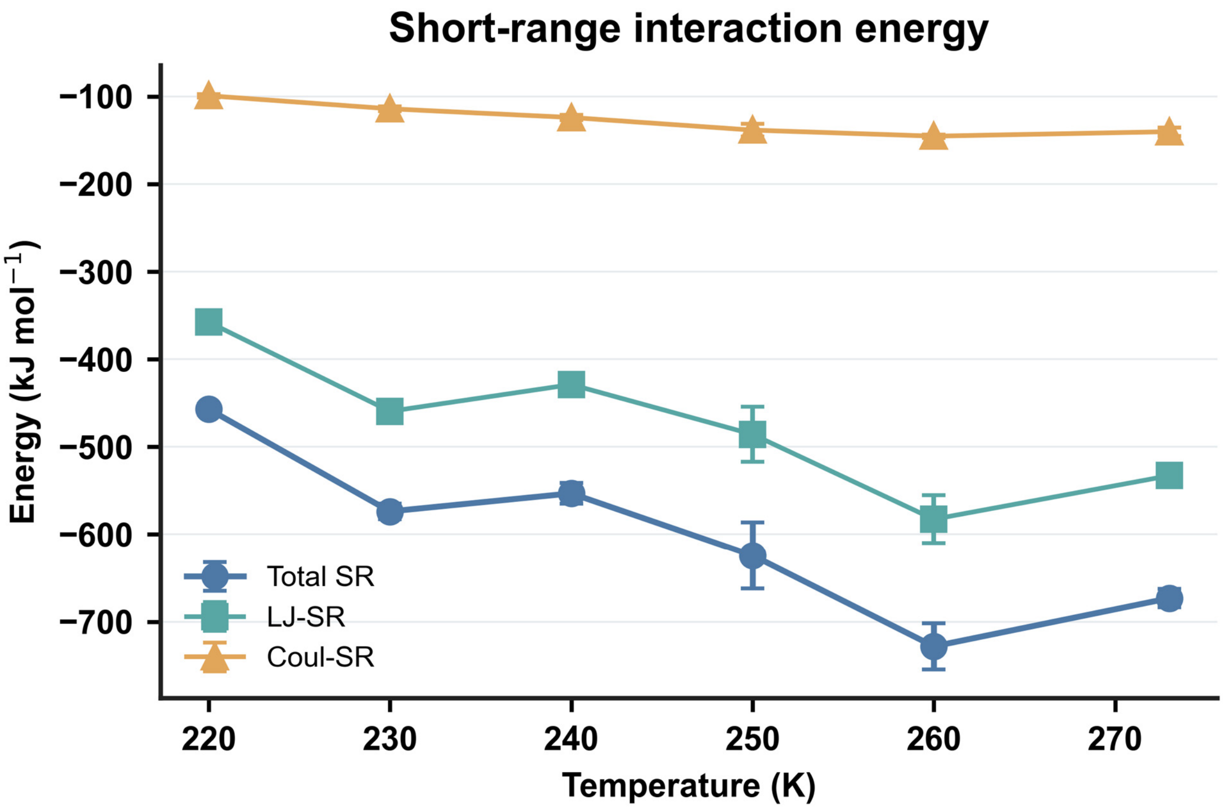

Figure S3. Short-range asphalt-ice interaction energy (Coul-SR + LJ-SR) across temperatures, recalculated from 5 ns trajectories. Error bars represent block SEM from the 1-5

ns analysis window and are not independent repeat-simulation error bars.

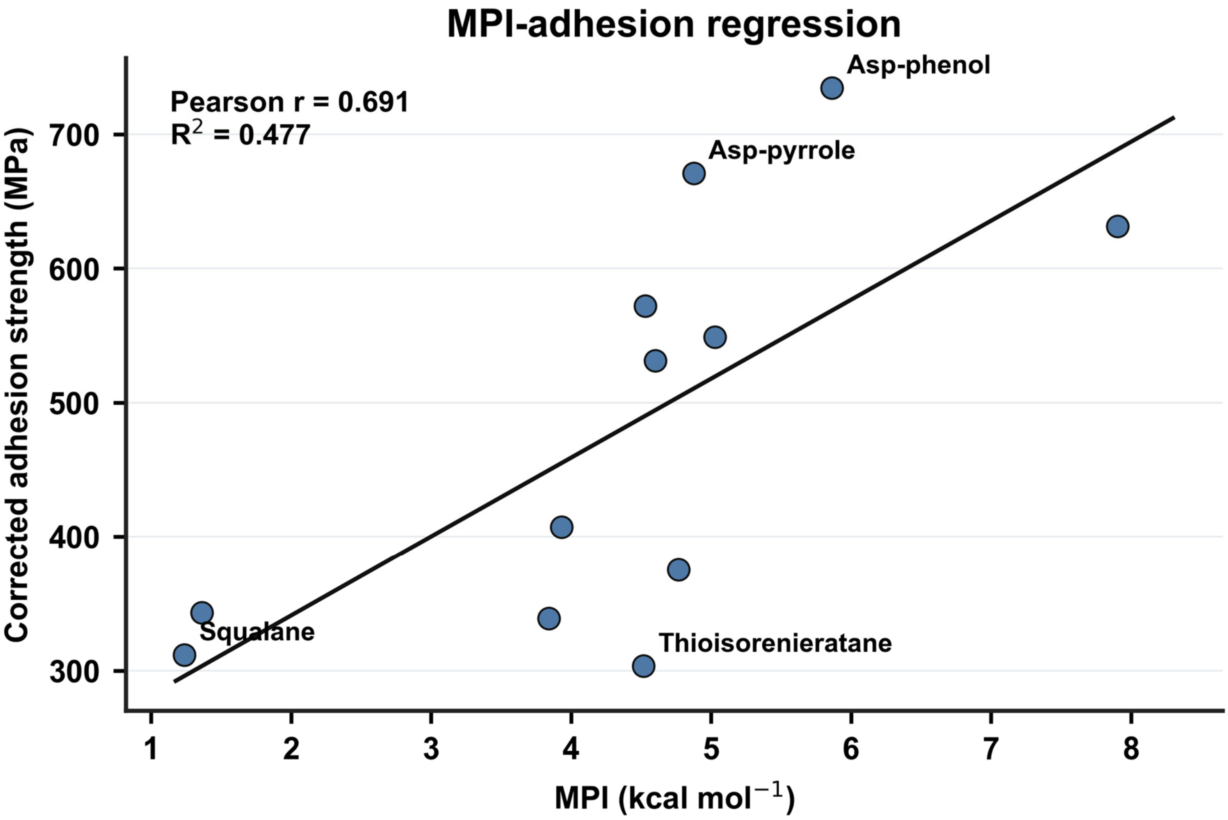

Figure S4. Exploratory association between the surface-electrostatic-potential molecular polarity index and molecular-scale nominal adhesion strength. The descriptor-level regression is reported as an association, not as a single-variable causal law.

Reference

[1] D.D. Li, M.L. Greenfield, Chemical compositions of improved model asphalt systems for molecular simulations, Fuel 115 (2014) 347-356.

[2] L. Zhang, M.L. Greenfield, Analyzing properties of model asphalts using molecular simulation, Energy Fuels 21 (2007) 1712-1716.

[3] J.L.F. Abascal, E. Sanz, R. Garcia Fernandez, C. Vega, A potential model for the study of ices and amorphous water: TIP4P/Ice, J. Chem. Phys. 122 (2005) 234511.

[4] M.J. Abraham, T. Murtola, R. Schulz, S. Pall, J.C. Smith, B. Hess, E. Lindahl, GROMACS: High

performance molecular simulations through multi-level parallelism from laptops to supercomputers, SoftwareX 1-2 (2015) 19-25.

[5] W.L. Jorgensen, D.S. Maxwell, J. Tirado-Rives, Development and testing of the OPLS all-atom force field on conformational energetics and properties of organic liquids, J. Am. Chem. Soc. 118 (1996) 11225-11236.

[6] T. Darden, D. York, L. Pedersen, Particle mesh Ewald: An N log(N) method for Ewald sums in large systems, J. Chem. Phys. 98 (1993) 10089-10092.

[7] B. Hess, H. Bekker, H.J.C. Berendsen, J.G.E.M. Fraaije, LINCS: A linear constraint solver for molecular simulations, J. Comput. Chem. 18 (1997) 1463-1472.

[8] Y. Jiao, Y. Yao, H. Qiu, H. Chen, Y. Wu, A molecular dynamics analysis of the thickness and adhesion characteristics of the quasi-liquid layer at the asphalt-ice interface, Materials 17 (2024) 1375. DOI: 10.3390/ma17061375.

[9] Y. Yao, Y. Jiao, W. Zhao, Effect of the experimental parameters on the ice-asphalt adhesion strength and corresponding theoretical model based on the energy-balanced principle, Cold Reg. Sci. Technol. 222 (2024) 104182. DOI: 10.1016/j.coldregions.2024.104182
